# Agreement Between a Mobile Self-Administered Comprehensive Geriatric Assessment Screening Tool and Geriatrician-Administered Assessment: Cross-Sectional Feasibility Study

**DOI:** 10.2196/90863

**Published:** 2026-09-21

**Authors:** Daniele Fabrino Cupertino Queirod De Oliveira, Ana Paula Cupertino, Samara Morais Silveira, Rebeca Costa Barbosa, Leonardo Spencer de Vasconcellos, Luiz Sérgio Fernandes

**Affiliations:** 1 Escola Superior de Ciências da Saúde (ESCS), Universidade do Distrito Federal (UnDF), Brasília, Brazil; 2 Unidade de Geriatria, Hospital de Base, Brasilia, Federal District, Brazil; 3 Instituto Hospital de Base do DF, Federal District, SMHS, Área Especial, Quadra 101, CEP 70.335-900, Brazil, +55 (61) 98307-4422; 4 Surgical Health Outcomes Research Enterprise, Departments of Internal Medicine and Cardiology, University of Rochester Medical Center, Rochester, NY, United States; 5 Hospital Sírio Libanes, Brasilia, Brazil; 6 Data Lab for Quality of Care and Outcomes Research (LaDaQCOR), Universidade Católica de Brasília (UCB), Brazil; 7 Aramari Apo Institute for Advanced Health Education and Research, Brasília, Brazil

**Keywords:** self-assessment, comprehensive geriatric assessment, mobile health, older adults, geriatric screening

## Abstract

**Background:**

Comprehensive geriatric assessment (CGA) is a widely recommended, multidimensional approach for guiding clinical decision-making and management in older adults. However, its implementation remains limited by a shortage of geriatric specialists, the complexity of geriatric care, and the increasing demands of an aging population. Mobile self-administered tools offer a potential strategy to expand access to multidimensional geriatric evaluation, particularly in resource-limited settings.

**Objective:**

This study aimed to evaluate the feasibility and agreement of a mobile self-administered CGA screening tool compared with geriatrician-administered assessments among inpatient and outpatient older adults in Brazil.

**Methods:**

This cross-sectional study included 80 older adults recruited from inpatient and outpatient geriatric clinics in Brasília, Brazil. Participants completed a mobile CGA, which incorporated validated instruments covering functional status (Vulnerable Elders Survey–13 [VES-13]), cognition (Cognitive Change Questionnaire–8 [CCQ-8]), depressive symptoms (5-item Geriatric Depression Scale [GDS-5]), nutritional status (Mini Nutritional Assessment [MNA]), frailty (G8 screening tool), social support (Gijón Scale), falls, and vision and hearing. Within 48 hours, a geriatrician independently performed a geriatrician-administered CGA using the same instruments. Agreement between the 2 assessment methods was evaluated using Wilcoxon signed-rank tests, Spearman correlation coefficients, intraclass correlation coefficients (ICCs), and Cohen κ coefficients for categorical variables.

**Results:**

Participants had a mean age of 70 (SD 7) years; 58.8% (47/80) were female, and 65% (52/80) had ≤8 years of education. The mean completion time for the self-administered CGA was 18.5 (SD 7.5) minutes. Most participants rated the tool as easy or very easy to use (65/80, 81.3%) and reported satisfaction with the assessment process (n=79, 98.8%). High concordance was observed between the self-administered and geriatrician-administered CGA versions for most domains. Cohen κ coefficients demonstrated strong agreement for falls (κ=0.878; *P*<.001), hearing impairment (κ=0.826), and vision impairment (κ=0.634). No significant differences were observed between the 2 assessment methods for functional status (VES-13), frailty screening (G8), depressive symptoms (GDS-5), and cognition (CCQ-8). Nutritional status and social vulnerability showed lower concordance than other CGA domains. Among inpatients, significant discrepancies were identified in both nutritional scores and Gijón social risk scores (*P*≤.05), with participants reporting greater perceived vulnerability. For outpatients, significant differences were observed for nutritional status (MNA; P=.02) and frailty screening (G8; P=.046).

**Conclusions:**

A mobile self-administered CGA demonstrated good feasibility, high user satisfaction, and substantial agreement with geriatrician-administered assessments across multiple geriatric domains. The tool may support geriatric screening and triage in settings with limited specialist availability, particularly when used as a complement to comprehensive clinical assessment.

## Introduction

The global aging population continues to place increasing demands on public health systems, requiring policy adaptations and reshaping social dynamics, a trend particularly evident in Brazil [1-3]. In the Brazilian federal district, favorable socioeconomic conditions have contributed to a higher-than-average proportion of older adults, including nearly 300 centenarians [4]. However, marked social inequalities between central and peripheral areas create unequal access to essential services such as geriatric care [5]. These disparities highlight the need for strategies tailored to diverse socioeconomic and geographic contexts, including the adoption of low-cost, scalable digital health tools adapted for older adults. Such technologies can improve resource efficiency, extend the reach of geriatric services for diagnosis and care management [6,7], and promote greater patient engagement in care.

As populations age, the demand for comprehensive geriatric care continues to increase. These needs often require interdisciplinary interventions, underscoring the importance of comprehensive geriatric assessment (CGA) as a structured, person-centered approach to guiding individualized care planning. Access to geriatric care, however, remains limited, particularly in low- and middle-income countries, due to the global shortage of geriatric specialists [6,8]. To address these needs, several validated screening instruments have been developed, including the Mini-Mental State Examination [9], the Activities of Daily Living scale [10], the Mini Nutritional Assessment (MNA) [11], the Gijón Social Support Scale [12], and the 5-item Geriatric Depression Scale (GDS-5) [13].

Although these instruments are valuable individually, they are most effective when integrated into a CGA, a multidimensional, interdisciplinary process that combines clinical, functional, cognitive, nutritional, and psychosocial data to guide care [14]. CGA has been widely recommended as an important approach to support clinical decision-making in older adults undergoing medical or surgical interventions, particularly in oncology and other complex care settings [15-17]. CGA has demonstrated effectiveness in improving risk stratification and clinical outcomes across various specialties, including oncology, orthopedics, and cardiology [16,18-21]. Despite its relevance to clinical care, CGA implementation is often hindered by the need for trained specialists, coordinated teams, and structured workflows [22].

To overcome these barriers, self-administered tools, particularly mobile-based apps, are emerging as promising alternatives to increase the reach of CGA [4,6,23-26]. These tools allow older adults to complete assessments independently, with results reviewed remotely by geriatricians, potentially expanding access to geriatric care in underserved areas. Early studies have demonstrated the feasibility and acceptability of these tools among highly educated, English-speaking populations. Previous studies have also demonstrated the feasibility of self-administered CGA tools, even among older adults with complex conditions such as cancer [20,27].

In Brazil, digital health transformation has expanded following the implementation of the Ministry of Health’s Digital Health Strategy, with further momentum driven by the COVID-19 pandemic [28-30]. Persistent structural barriers, including low income, older age, limited education, and inadequate internet access, might hinder the equitable uptake of these technologies [30-33]. Despite these challenges, digital tools are increasingly recognized as essential in geriatric care for promoting health maintenance, functional independence, and quality of life in Brazil [24-26]. This study aims to evaluate the feasibility of a mobile self-administered CGA tool for older adults and to compare its performance with that of geriatrician-administered assessments among inpatient and outpatient older adults in Brazil.

## Methods

### Study Design and Setting

This was a cross-sectional study conducted between March and October 2024 in both hospital inpatient and outpatient settings in Brasília, the capital of Brazil and the administrative center of the Federal District. Data collection took place at 2 sites: the inpatient geriatric care unit at the *Hospital de Base do Distrito Federal* (HBDF), a large public hospital integrated into Brazil’s Unified Health System (*Sistema Único de Saúde* [SUS]), and the Integrated Outpatient Center (*Centro Integrado Ambulatorial* [CIA]) of the Catholic University of Brasília (*Universidade Católica de Brasília* [UCB]).

### Ethical Considerations

The study protocol was approved by the research ethics committee through the Brazilian CEP/CONEP (*Comitê de Ética em Pesquisa*–*Comissão Nacional de Ética em Pesquisa*) system (6.567.586). All participants provided written informed consent prior to enrollment. Data were anonymized and stored in a secure cloud-based system with encrypted storage and restricted access limited to authorized researchers. Participants received no financial compensation for participation.

### Participants and Procedures

Participants were aged ≥60 years, in accordance with Brazilian and World Health Organization guidelines. A trained research assistant screened potentially eligible participants in both inpatient and outpatient settings and obtained written informed consent. Inpatients were identified through daily screening of vascular surgery admissions, whereas outpatients were recruited during routine geriatric clinic visits.

Cognitive eligibility was established through medical record review, clinician judgment, and the participant’s ability to independently complete the assessment and provide informed consent. Individuals with severe cognitive impairment, illiteracy, or acute clinical instability were excluded.

Participants completed the self-administered CGA on a touch-screen tablet, with research staff available to provide technical clarification if needed. After completing the CGA, participants answered a standardized usability questionnaire assessing ease of use, navigation, and clarity. Within 48 hours, a geriatrician performed the geriatrician-administered assessment using the same instruments. Data were stored in a secure cloud-based system with encrypted storage and restricted access. No major clinical interventions, including surgery, occurred between the self-administered and geriatrician-administered assessments.

### Mobile Tool Development and Design

A mobile CGA tool was implemented using a structured Google Forms (Alphabet Inc) interface optimized for tablet-based use. The design followed geriatric-friendly principles, incorporating simplified language and touch-responsive options to facilitate independent navigation by older adults. All instruments were based on validated versions adapted for the Brazilian context. Data were stored in an encrypted cloud-based system with restricted access. Prior to data collection, the tool was tested by the research team to ensure usability and appropriate navigation flow.

Selected items from the CONSORT-EHEALTH (Consolidated Standards of Reporting Trials of Electronic and Mobile Health Applications and Online Telehealth) checklist were used to guide reporting of the digital tool, including its usability features and implementation workflow.

### Measures

The assessment incorporated validated measures in Portuguese, aligned with the domains recommended by the Brazilian Society of Geriatrics and Gerontology for CGA. The CGA evaluated functional status (Vulnerable Elders Survey–13 [VES-13]), cognition (Cognitive Change Questionnaire–8 [CCQ-8]), emotional health (GDS-5), nutritional status (MNA), fall risk (self-reported falls in the past 6 months), social support (Gijón Scale), and frailty (G8 screening tool). Vision and hearing impairment were assessed through self-reported structured questions. All instruments were applied in their validated Portuguese versions. Participants completed the digital CGA on a tablet designed according to accessible design principles, including large fonts, high contrast, intuitive icons, and a light background. Demographic variables included sex, age, weight, height, BMI, marital status, and education level.

Feasibility outcomes included assessment completion time, participant satisfaction, and perceived ease of use of the digital tool.

### Statistical Analysis

Sample size was determined based on the precision required to estimate the intraclass correlation coefficient (ICC). Assuming an expected ICC of 0.80, a sample of 80 paired assessments was estimated to provide approximately 80% assurance that the lower bound of the 1-sided 95% CI would remain >0.70, a threshold considered indicative of good agreement. This approach followed the precision and assurance framework described by Zou [34].

Statistical assumptions were initially checked, including the presence of outliers, missing data, and normality. Because the data were not normally distributed, nonparametric tests were applied, following the recommendations of Tabachnick and Fidell [35]. Spearman rank correlation was used to examine agreement between self-administered and geriatrician-administered assessments. Wilcoxon signed-rank tests were used to compare paired scores overall and by subgroup (inpatients vs outpatients). For categorical variables such as history of falls, hearing impairment, and vision impairment, Cohen κ coefficient was used to assess item-level agreement, as it provides a more robust estimate than simple concordance percentages by adjusting for chance agreement [36,37]. Between-group comparisons by sex and care setting used the Mann-Whitney *U* test. Spearman rank correlations also examined associations between education and assessment or usability variables, including completion time and satisfaction. All analyses were 2-tailed, and *P*<.05 was considered statistically significant. Data were processed using SPSS (version 27; IBM Corp).

## Results

A total of 80 participants aged 63 to 77 (mean 70, SD 7) years were included in the study. Of these participants, 58.8% (n=47) were women, and the majority were married. Most participants were of low socioeconomic status: two-thirds (n=52, 65%) had ≤8 years of formal education, and 68% (n=55) reported earning a minimum wage of US $320 per month. The average time to complete the CGA was 18.5 (SD 7.5) minutes. Notably, 99% (n=79) of participants reported being satisfied or very satisfied with the CGA, and 81.3% (n=65) found it easy or very easy to complete the mobile tool (Table 1).

**Table 1.** Participant characteristics and feasibility outcomes (N=80).

Variables	Participants
Age (y), mean (SD)	70 (7)
Sex, n (%)	
Female	47 (58.8)
Male	33 (41.2)
Educational level (y), n (%)	
1‐4	28 (35)
5‐8	24 (30)
9‐12	17 (21.2)
13‐16	11 (13.8)
Marital status, n (%)	
Single	9 (11.2)
Married	44 (55)
Widowed	17 (21.2)
Divorced	10 (12.5)
Clinical setting, n (%)	
Outpatient	36 (45)
Inpatient	44 (55)
Assessment completion time (min), mean (SD)	18.5 (7.5)
Satisfaction, n (%)	
Very dissatisfied	0 (0)
Dissatisfied	1 (1.2)
Satisfied	14 (17.5)
Very satisfied	65 (81.2)
Ease of use, n (%)	
Very difficult	4 (5)
Difficult	11 (13.8)
Easy	28 (35)
Very easy	37 (46.2)

To evaluate the feasibility of older adults using the mobile CGA tool, we compared assessments completed independently by older adults with those conducted by a professional. As hypothesized, a high level of concordance was observed between self-administered and geriatrician-administered assessments. Across all instruments, VES-13 (*P*=.76), MNA (*P*=.12), GDS (*P*=.32), CCQ-8 (*P*=.10), G8 (*P*=.17), and Gijón Scale scores (*P*=.36) showed no statistically significant differences in score distributions according to the Wilcoxon signed-rank test (Table 2).

**Table 2.** Agreement between self-administered and geriatrician-administered comprehensive geriatric assessment classifications (N=80).

Instruments and categories	Geriatrician-administered, n (%)	Self-administered, n (%)	*P* value^a^
Vulnerable Elders Survey–13			.76
Not vulnerable	63 (78.8)	62 (77.5)	
Vulnerable	17 (21.2)	18 (22.5)	
Mini Nutritional Assessment			.12
Malnourished	7 (8.8)	12 (15)	
At risk of malnutrition	39 (48.8)	37 (46.2)	
Normal nutritional status	34 (42.5)	31 (38.8)	
5-item Geriatric Depression Scale			.32
No depression	50 (62.5)	47 (58.8)	
Probable depression	30 (37.5)	33 (41.2)	
Cognitive Change Questionnaire–8			.10
Normal	17 (21.2)	14 (17.5)	
Cognitive impairment	55 (68.8)	52 (65)	
Dementia	8 (10)	14 (17.5)	
G8 screening tool			.17
Normal	26 (32.5)	21 (26.2)	
Abnormal	54 (67.5)	59 (73.8)	
Gijón Scale^b^			.36
Low social risk	5 (6.2)	3 (3.8)	
Moderate social risk	13 (16.2)	13 (16.2)	
High social risk	62 (77.5)	64 (80)	

a *P* values were calculated using the Wilcoxon signed-rank test for paired comparisons.

b Higher Gijón Scale scores indicate greater social vulnerability.

Self-administered anthropometric indicators also showed concordance with geriatrician-administered assessments for weight (outpatients: *P*=.18; inpatients: *P*=.83), height (outpatients: *P*=.27; inpatients: *P*=.07), and BMI (outpatients: *P*=.26; inpatients: *P*=.68; Table 3). Supporting these findings, Cohen κ coefficients demonstrated substantial agreement in objectively verifiable domains, including history of falls (κ=0.878), hearing impairment (κ=0.826), and visual impairment (κ=0.634), all with *P*<.001 (Table 4). These results underscore the reliability of self-administered data in key domains of geriatric assessment and support the use of mobile self-administered tools as a viable and acceptable alternative for evaluating older adults.

**Table 3.** Comparison between self-administered and geriatrician-administered assessments by clinical setting.

Variables	Outpatient self-administered, median (IQR)	Outpatient geriatrician-administered, median (IQR)	*P* value	Inpatient self-administered, median (IQR)	Inpatient geriatrician-administered, median (IQR)	*P* value
Weight	67 (59.5-74.0)	67.3 (59.5-75.0)	.18	68 (62.0-75.0)	68 (62.0-75.0)	.83
Height	1.6 (1.5-1.6)	1.6 (1.5-1.6)	.27	1.7 (1.6-1.7)	1.6 (1.6-1.7)	.07
BMI	27.4 (24.4-29.7)	27.3 (24.0-29.7)	.26	24.8 (23.0-26.8)	25 (22.8-28.2)	.68
Mini Nutritional Assessment	11.4 (9.5-14.0)	11.8 (10.0-14.0)	.02	9 (8.0-11.0)	10 (9.0-12.0)	.02
Gijón Scale	11.5 (9.0-12.5)	11.3 (9.0-12.0)	.21	13 (11.0-15.0)	11 (10.0-13.0)	.002
5-item Geriatric Depression Scale	1.5 (0.0-2.0)	1.5 (0.0-2.0)	.52	1 (1.0-2.0)	1 (1.0-2.0)	.54
Cognitive Change Questionnaire–8	2.0 (0.5-3.0)	1.5 (0.0-2.5)	.17	1 (0.0-3.0)	1 (0.0-2.0)	.34
G8 screening tool	13.5 (12.0-16.0)	14.0 (13.0-16.0)	.046	12 (10.0-13.5)	13 (11.0-14.0)	.05
Vulnerable Elders Survey–13	1.0 (0.0-1.0)	1.0 (0.0-1.0)	>.99	1 (0.0-5.0)	0 (0.0-4.5)	.15

**Table 4.** Agreement for categorical clinical variables between self-administered and geriatrician-administered assessments (N=80)^a^^,^^b^.

Variables	Self-administered, n (%)	Geriatrician-administered, n (%)	Cohen κ	*P* value
Falls	23 (28.8)	23 (28.8)	0.878	<.001
Hearing impairment	24 (30)	26 (32.5)	0.826	<.001
Vision impairment	59 (73.8)	57 (71.2)	0.634	<.001

a Cohen κ coefficients were used to evaluate agreement between self-administered and geriatrician-administered assessments.

b The self-administered and geriatrician-administered assessments were performed in the same 80 participants; therefore, the counts and percentages across these columns are not mutually exclusive and should not be summed.

There was concordance in most CGA scores between self-administered and geriatrician-administered assessments for older adults in both inpatient and outpatient settings. No statistically significant differences were observed between self-administered and geriatrician-administered scores for depressive symptoms (GDS: outpatients, *P*=.52; inpatients, *P*=.54), cognitive function (CCQ-8: outpatients, *P*=.17; inpatients, *P*=.34), or multidimensional vulnerability assessed with the VES-13 (outpatients, *P*>.99; inpatients, *P*=.15; Table 3). These findings support the feasibility of using the mobile CGA tool in both hospitalized and clinic-based older adults.

The only statistically significant discrepancies were observed in MNA nutritional scores, with participants consistently reporting lower scores than professionals for both outpatients (*P*=.02) and inpatients (*P*=.02). In other words, older adults rated themselves as having greater nutritional impairment than was identified by health care professionals in both settings. G8 frailty scores showed no significant difference among inpatients (*P*=.05), whereas a small difference was observed among outpatients (*P*=.046), with professionals rating greater vulnerability than participants in both settings (Table 3). In the social domain, the Gijón social risk score showed a significant discrepancy in inpatient settings (*P*=.002), where participants reported higher levels of social risk than geriatricians.

Considering educational level, professionals rated social risk as higher among individuals with lower education. In contrast, older adults’ self-assessed social risk was not associated with educational level. A similar pattern was observed for CCQ-8 cognitive scores: lower educational attainment was associated with poorer cognitive performance in geriatrician-administered assessments, with no statistically significant association observed for the self-administered tool. As expected, participants with lower educational attainment required more time to complete the self-assessment (*ρ*=–0.266; *P*=.02) and reported lower perceived ease of use (ρ=0.510; *P*<.01). Satisfaction with the tool was high across all educational levels and was not significantly associated with education (*ρ*=.058; Table 5).

**Table 5.** Correlation between educational level, usability variables, and assessment completion time^a^.

Variables	Assessment completion time (min)		Satisfaction		Ease of use	
	ρ	*P* value	ρ	*P* value	ρ	*P* value
Satisfaction	−0.112	.32	—^b^	—	—	—
Ease of use	−0.079	.45	0.281	.01	—	—
Educational level	−0.266	.02	0.058	.61	0.510	<.01

a ρ indicates the Spearman rank correlation coefficient.

b Em dashes indicate self-correlations or duplicate pairwise correlations that are not reported.

## Discussion

### Principal Results

This study demonstrates that a mobile, self-administered geriatric screening tool based on the domains of the CGA is a feasible, acceptable, and reliable method for screening older adults in both inpatient and outpatient settings in Brazil. It is important to emphasize that this mobile tool should be interpreted as a screening and triage instrument rather than a replacement for the full CGA. Its main value lies in helping to save specialist time, broaden access, and identify older adults who are most likely to benefit from a complete CGA. Our findings support the utility of this mobile tool as a screening gateway to the CGA, complementing but not substituting for the clinical comprehensiveness of professional assessments.

High concordance was observed between self-administered and geriatrician-administered assessments across multiple objective domains, including hearing, vision, and history of falls. The use of Cohen κ strengthened the reliability analysis in these categorical domains, as this measure adjusts for agreement expected by chance, reinforcing the robustness of concordance in falls, hearing, and vision assessments.

### Comparison With Prior Work

Our findings are consistent with prior studies demonstrating the feasibility and reliability of self-administered geriatric assessment tools, particularly those delivered through digital platforms and supported by user-centered design principles. Previous research has shown that mobile or electronic CGA tools can produce results comparable to clinician-administered assessments, even among older adults with complex health conditions or those from vulnerable populations [23,24,27,38]. Prior studies have similarly shown that mobile platforms, culturally adapted instruments, and user-centered design approaches can enhance accessibility, reliability, and clinical utility.

Despite these promising results, discrepancies emerged in more subjective domains, particularly nutritional status (MNA) and social vulnerability (Gijón Scale). In both inpatient and outpatient settings, participants rated their nutritional status as poorer than geriatricians, which may reflect self-perceived vulnerability influenced by appetite changes, undocumented weight loss, and the cumulative impact of chronic conditions—factors that may not always be captured in professional evaluations. In the social domain, inpatients reported higher levels of social risk than were perceived by professionals. This likely reflects feelings of isolation, loss of autonomy, and heightened vulnerability associated with hospitalization, experiences that are not always evident in an objective clinical assessment. Such findings suggest that self-reports can capture subjective perceptions and psychosocial stressors that may remain invisible in geriatrician-administered assessments [36,39].

### Educational Level and Usability Considerations

Educational level also influenced a few outcomes. Geriatricians’ social risk ratings increased with lower educational attainment, whereas older adults’ self-assessed social risk was not associated with education. Similarly, lower educational attainment correlated with poorer cognitive scores on the CCQ-8 in geriatrician-administered assessments, with only marginal significance in self-administered results. As expected, participants with fewer years of education required more time to complete the self-assessment and reported lower ease of use, yet satisfaction remained high across all educational groups. These findings highlight the importance of inclusive and user-centered interface design, including large fonts, high-contrast layouts, simplified language, and intuitive navigation, to reduce digital barriers for older adults with lower literacy or limited technology experience. Additional accessibility features, such as audio narration, multimedia guidance, and optional user support, may further enhance usability, particularly among individuals with very low educational attainment or limited digital familiarity.

### Implications for Health Systems and Practice

From a systems perspective, integrating mobile CGAs into electronic health records (EHRs) and care coordination platforms could enable automated risk stratification, population health management, and timely referrals to interdisciplinary teams. In low- and middle-income countries, this approach may improve care continuity while reducing demands on scarce geriatric specialists. In our study, the average completion time of <20 minutes, combined with high satisfaction ratings, supports the feasibility of incorporating the self-administered CGA into routine workflows. This is particularly relevant within Brazil’s Unified Health System (SUS), where specialist availability is limited. Previous research has shown that digital CGAs can streamline clinical encounters without compromising care quality [24] and that structured electronic tools can enhance diagnostic accuracy and geriatrician acceptance [40].

### Limitations

This study has several limitations. It focused primarily on urban, literate older adults, which may limit generalizability to rural populations, individuals with low literacy, or those with moderate to severe cognitive impairment. Future adaptations should explore simplified interfaces, audio guidance, caregiver-assisted administration, and other accessibility strategies to improve the inclusion of socially, educationally, and cognitively vulnerable older adults.

A prospective screening log documenting all potentially eligible individuals, refusals, and reasons for nonparticipation was not systematically maintained. Therefore, participation rates and the potential magnitude of selection bias could not be fully assessed.

Because the self-administered assessment was completed before the geriatrician-administered evaluation, an order effect may have artificially increased agreement between methods. However, geriatricians performing the professional assessments did not have access to participants’ self-administered responses, reducing the likelihood of direct observer bias. Although assessments were conducted within 48 hours, minor clinical fluctuations may still have influenced some responses.

The cross-sectional design allowed the evaluation of agreement and usability but not predictive validity. Longitudinal studies are needed to determine whether self-administered CGA scores are associated with clinically meaningful outcomes such as hospital readmissions, functional decline, institutionalization, and mortality.

Finally, because multiple comparisons were performed across CGA domains and care settings, the possibility of type I error should be considered. Given the exploratory nature of the study, no formal correction for multiple comparisons was applied; therefore, domain-specific findings should be interpreted with caution.

### Conclusions

The mobile, self-administered CGA demonstrates strong potential to expand access to geriatric screening and triage, particularly in settings with limited specialist availability. As populations age and health care systems face increasing demands, such tools may support early detection, care coordination, and more equitable outcomes, especially in low- and middle-income countries.

By integrating patient-reported data into digital health systems, these tools enhance person-centered, data-informed care. High concordance in objective domains supports the reliability of the self-administered tool, while the link between perceived vulnerability and adverse outcomes highlights the value of including subjective measures when they are culturally adapted.

Importantly, this tool is not a substitute for clinical evaluation but rather a complement to existing workflows and may ultimately enhance patient engagement in care.

In summary, the self-administered mobile tool should be understood as a screening and triage instrument that complements, rather than replaces, the full CGA. By integrating objective measures with patient-reported perceptions, it has the potential to expand access to geriatric screening, reduce the burden on specialists, and support clinical decision-making and public health strategies aimed at healthy aging, particularly in resource-limited health systems.

## Abbreviations

CCQ-8: Cognitive Change Questionnaire–8
CEP: *Comitê de Ética em Pesquisa*
CGA: comprehensive geriatric assessment
CIA: *Centro Integrado Ambulatorial*
CONEP: *Comissão Nacional de Ética em Pesquisa*
CONSORT-EHEALTH: Consolidated Standards of Reporting Trials of Electronic and Mobile Health Applications and Online Telehealth
EHR: electronic health record
GDS-5: 5-item Geriatric Depression Scale
HBDF: *Hospital de Base do Distrito Federal*
ICC: intraclass correlation coefficient
MNA: Mini Nutritional Assessment
SUS: *Sistema Único de Saúde*
UCB: *Universidade Católica de Brasília*
VES-13: Vulnerable Elders Survey–13
